# Bicycle Facilities That Address Safety, Crime, and Economic Development: Perceptions from Morelia, Mexico

**DOI:** 10.3390/ijerph15010001

**Published:** 2017-12-22

**Authors:** Inés Alveano-Aguerrebere, Francisco Javier Ayvar-Campos, Maryam Farvid, Anne Lusk

**Affiliations:** 1 Instituto de Investigaciones Económicas y Empresariales, Universidad Michoacana de San Nicolás de Hidalgo, 58040 Morelia, Mexico; inesalag@gmail.com (I.A.-A.); franciscoayvar@hotmail.com (F.J.A.-C.); 2 Harvard T. H. Chan School of Public Health, 655 Huntington Avenue, Building II Room 314, Boston, MA 02115, USA; mfarvid@hsph.harvard.edu

**Keywords:** bicycle infrastructure, bicycling choice, crash safety, crime lowering, economic development, developing nation

## Abstract

México is a developing nation and, in the city of Morelia, the concept of the bicyclist as a road user appeared only recently in the Municipal Traffic Regulations. Perhaps the right bicycle infrastructure could address safety, crime, and economic development. To identify the best infrastructure, six groups in Morelia ranked and commented on pictures of bicycle environments that exist in bicycle-friendly nations. Perceptions about bike paths, but only those with impossible-to-be-driven-over solid barriers, were associated with safety from crashes, lowering crime, and contributing to economic development. Shared use paths were associated with lowering the probability of car/bike crashes but lacked the potential to deter crime and foster the local economy. Joint bus and bike lanes were associated with lower safety because of the unwillingness by Mexican bus drivers to be courteous to bicyclists. Gender differences about crash risk biking in the road with the cars (6 best/0 worst scenario) were statistically significant (1.4 for male versus 0.69 for female; *p* < 0.001). For crashes, crime, and economic development, perceptions about bicycle infrastructure were different in this developing nation perhaps because policy, institutional context, and policing (ticketing for unlawful parking) are not the same as in a developed nation. Countries such as Mexico should consider building cycle tracks with solid barriers to address safety, crime, and economic development.

## 1. Introduction

Low-income families in Mexico spend as much as 50% of their household income on transport [1]. In Mexico, the bicyclist was only recently (January 2014) incorporated in the Municipal Regulation of Traffic and Roads as a road user, along with the validation of the need to promote this mode of transport [2]. In Morelia, Mexico, the capital of Michoacán state, more than 50% of the trip distances are less than 3 miles. Morelia has a temperate climate with relatively flat terrain and travel is principally by public transport (40%) followed by walking (35%), private vehicle (21%) and cab (3%) (commutes by bike had not been included in this collection of data) [3]. Even with these conditions, Morelia has high levels of vehicle traffic, pollution from mobile sources [4], and poor quality infrastructure for all the users of the road. 

Compared with walking, bicycling is an effective means of travel due to speed, distance covered, and destinations reached [5,6,7,8,9]. Stakeholders, academics and non-governmental organizations are looking at ways to increase bicycling as everyday travel to cover the shorter distances typically reached by transit or car [10,11,12,13,14,15,16,17,18,19]. Cycling as a feeder mode [20] and bike sharing systems [21] are also being explored to foster bicycling. 

Research conducted in New Zealand revealed that if, over the next 40 years, main roads have the best-practice bicycle facilities with physical separation from cars and local streets have low speeds, the benefits would be 10 to 25 times higher than the costs [22]. Cycle tracks provide separation from motor vehicles, a feature most preferred by female, child, and senior cyclists [16,18,23,24,25,26,27]. While bicycle facilities in The Netherlands, Denmark, the U.S., Canada, and China are extensively studied, bicycle facilities in Mexico have received less attention. As in other developing nations, the Mexican stakeholders are still focusing on the automobile.

In Morelia, the 19th largest metropolitan area in Mexico, policies have ignored cycling and walking as forms of transportation. Currently, 40% of the road injuries and deaths are pedestrians and cyclists, affecting mostly the low-income population [28,29]. Typical documents about the municipal infrastructure do not mention the word “bicycle” [30]. Due to the history of little government recognition of bicyclists, lack of safety and prejudice against cyclists (bicyclists are perceived as not having money for public transit or car ownership), residents in Morelia have been less willing to use bicycles as a means of transportation. 

In addition to taking care of modern health risks, such as physical inactivity, obesity and road injuries, Mexico’s stakeholders have also been trying to address crime and economic development. According to INEGI [31], the crime rate in México, which primarily includes assault, burglary, kidnapping, and homicide, has increased steadily since 2005. Criminal behavior is associated with certain built environments because an opportunity for crime can be enabled/discouraged in different urban forms [32,33,34,35,36,37,38]. The infrastructure does not cause the crime, but the infrastructure can present opportunities in a society affected by systemic hierarchical issues such as economic inequality. Infrastructure for transportation is a central part of any town or city environment and therefore a place where crime can be committed [39].

At the same time that crime has risen in Mexico, economic development has declined [40]. Having a deficient mobility/accessibility policy coupled with socio-economic hindrances could be related to the downturn in the economic growth of cities [41,42,43], further expanding the gap between rich and poor. An automobile-focused built environment hinders economic development [44]. Travel time reductions, from switching mode of transport, and cost-savings, from less expensive forms of transport, could provide significant economic benefits [42].

Crime Prevention Through Environmental Design” (CPTED) [36,45] and “Fixing Broken Windows” [46] theories have lessened the perception and existence of crime by cleaning up the environment and having eyes on the street [35]. Positive cues in the environment foster normative and respective behavior [47]. Transportation officials introduce positive cues for behavior when they install well-ordered stenciled barrier-protected cycle tracks bordered by landscaping. 

For economic benefits, stores profit after the nearby installation of safe bike locations (bike paths, racks, etc.) [48,49,50]. Compared with people who commute by car or transit, people who use the bike for transportation spend considerably less money on their daily travels, stop more frequently to shop, and spend more monthly. The bike environment also attracts non-cyclists, thus increasing clientele and fostering economic development.

Identification of the most beneficial bicycle infrastructure for safety, crime, and economic development is necessary because many cities have installed inadequate bicycle infrastructure or installed ideal bicycle infrastructure, such as a cycle track, and then never created a network of cycle tracks. In the U.S., many cities adopted the principles in “Complete Streets” in which a sharrow or bike lane was painted beside parked cars to accommodate bicyclists. Later research demonstrated the lack of safety of a sharrow and a painted bike lane compared with a cycle track [51]. A thorough analysis of cycle tracks throughout the U.S. suggested that cycle tracks were safer than other bicycle infrastructure [16]. The Federal Highway Administration (FHWA) published a document that identified the safety and preference of protected bike lanes (cycle tracks) over other bicycle infrastructure [52]. While side streets with less vehicular traffic, termed “low stress routes”, can serve as alternative bicycle routes [53], in a community with a higher rate of crime, isolated side streets might be less safe.

Provision of the right bike environment might result in fewer road injuries, lowered crime, and improved economic development. Yet to effect change, policymakers and city planners need to know a population’s perceptions about different bicycle environments. In nations with an established bike culture, such as The Netherlands, the U.S., and Canada, simple cues in the built environment are indicators for the correct places for drivers and cyclists. Due to culture, level of education, and a just-recently emergent bicycling history, individuals in developing nations, like Mexico, may perceive modest cues (painted lines, low rubber islands, or plastic delineator posts) as insufficient indicators that they should not drive or park on the places set aside for bicyclists. Therefore, this research asked individuals in groups (Phase One) to complete a survey and indicate how different bicycle environments are perceived by populations in a developing nation in relation to: (a) lowering car/bike crashes; (b) lowering crime; and (c) increasing economic development. This research further asked the individuals in the same groups (Phase Two) whether the perceptions of bicycle environments that exist in developed nations would be understood and respected in the same way by residents in a developing nation.

## 2. Materials and Methods

Six groups of individuals in Morelia, Mexico volunteered to participate in a Visual and Verbal Preference Survey. In the small, intimate groups, forty-three participants among the six groups shared their opinions while enjoying a free dinner. In Phase One of the dinner-evening, the survey included places for the participants to indicate their perception about the pictures of different bicycle environments related to the possibilities of crash, crime and economic development. A pilot test demonstrated that 30 images were too many, so only 22 pictures were included. This allowed time for the quantitative ranking and qualitative comments. Each picture of the bicycle environment was projected onto the screen until every one of the participants had ranked each picture for the three categories without sharing their perceptions with others (between one and three minutes each slide). For the Phase Two portion of the dinner-evening, the participants discussed their perceptions related to crash, crime and economic development while looking at the pictures again (between two and four minutes each). All comments about each bicycle environment were audio recorded. Qualitative comment analysis provided descriptions about each of the different bicycle facilities regarding safety, crime, and economic development. The Phase One data were analyzed to assess and compare with the Phase Two group comments.

### 2.1. Location and Study Population

**Figure 1:**
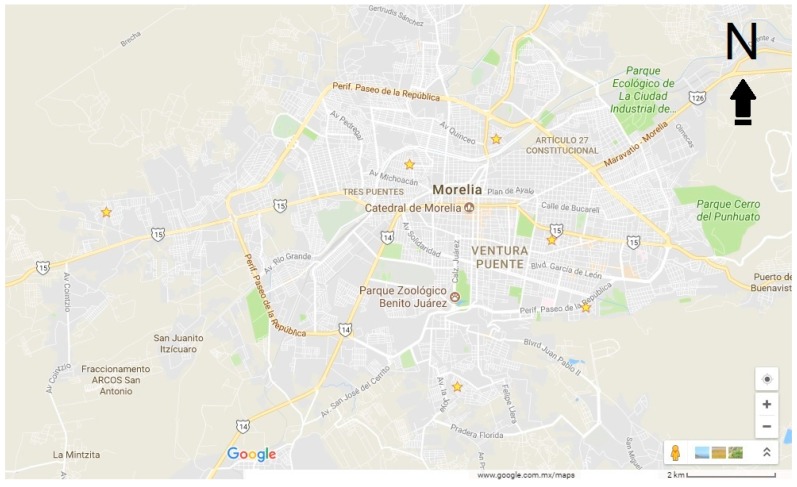
Location of the randomly selected neighborhoods.

The metropolitan area of Morelia, Mexico is comprised of more than 800,000 inhabitants, with a relatively low population density of 570 persons per square kilometer [54]. Morelia is the most populated and extensive city of the entity and represents 17.25% of the total population of Michoacán. In Morelia, invitations went by mail to four hundred and forty randomly selected households from six neighborhoods. Neighborhoods (Figure 1) where selected randomly from the city’s water supplier list, because it is more complete than the lists from the telephone or energy supplier. A broad social mix does not exist in most of the neighborhoods. Dinner locations included neighborhood public schools, public health community clinics, and area restaurants. These different restaurant locations would capture diverse socio-economic populations. From the completed surveys, the socioeconomic level of the participants was identifiable as being below or above the median. Participants did not have to reveal their usual travel mode because the aim was for the participant to picture him/herself traveling by bike.

The invitation explained that the participant would be given dinner and be asked to complete a survey to indicate their positive and negative perceptions about places to bicycle. The invitations underscored that the intention of the survey was to improve quality of life in Morelia. Included on the survey were the identity and qualifications of the interviewer. All of the locations chosen for the surveys had appropriate light, space, comfort, quiet and privacy and were close to participant’s residences.

### 2.2. Pictures of Bicycle Infrastructure Types

**Figure 2:**
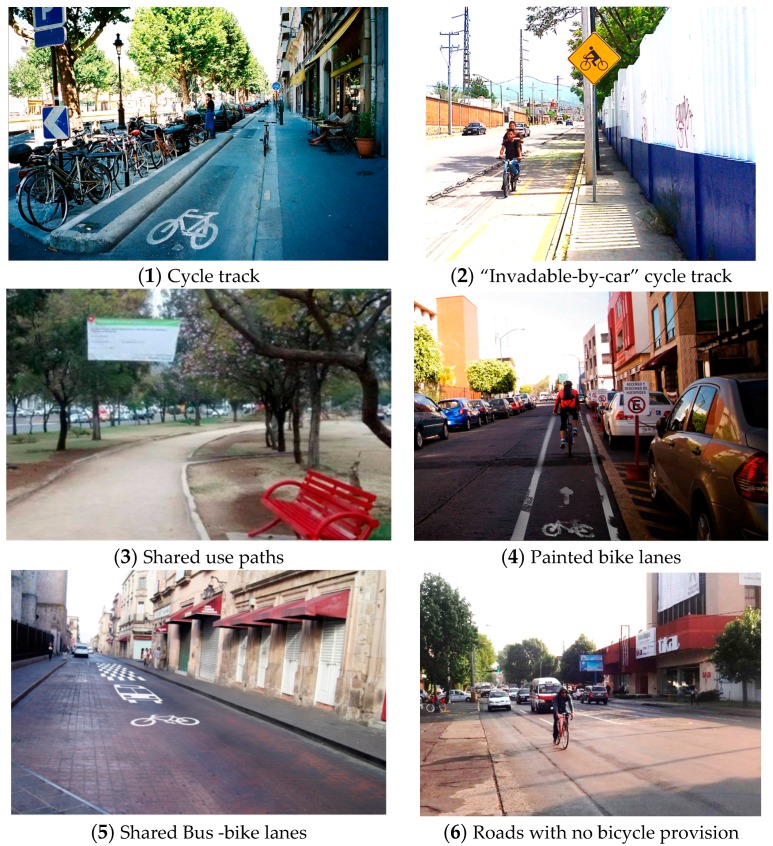
Types of bicycle environments included.

Pictures selected came from the authors’ private collection of bicycle environments worldwide while others were prepared for the survey to match the typical environment in Mexican cities. All of the pictures showed daytime light and good weather. Each picture contained very few to no bicyclists and an environment familiar to individuals in Mexico, e.g., no foreign traffic signs. The final survey contained 22 pictures. Several examples of the following types of bicycle environments were included (Figure 2):

(1) Cycle tracks—one and two way. Cycle tracks that have a physical barrier not easily driven over by vehicles. “Fortified areas with asphalt… A curb is placed on the roadway side as well as the sidewalk side” [55]. Separation of motorized and bicycle traffic [56].
(2) “Invadable by car” cycle tracks. Cycle tracks demarcated with low markers including low plastic curbs easily driven over by vehicles.
(3) Shared use paths. Park setting multi-use paths shared by different types of users (SHUP).
(4) Painted bike lanes that are between the sidewalk curb and moving cars or between parallel-parked cars and moving cars. Bicycle lanes are a portion of the roadway designated for preferential use by bicyclists. They are one-way facilities that typically carry bicycle traffic in the same direction as adjacent motor vehicle traffic [57].
(5) Bus and Bike Lanes. Sections of streets that buses and bicyclists share. Mexico has discussed allowing people on bicycles to ride on the bus rapid transit lanes.
(6) Roads with no bicycle provision. Roads with high traffic, downtown streets, and neighborhood streets on which there is no paint or provision for bicyclists.

### 2.3. Survey Questionnaire and Qualitative Comments

To ascertain perceptions about bicycle environments, we assembled groups of volunteers and used a Visual and Verbal Preference Survey (Phase One: survey; Phase Two: group discussions). All groups where led by one of the authors, who holds a Masters Degree in Public Health and Masters Degree in Applied Psychology and had prior experience in surveys and focus groups. She coded the data as well. Her occupation at the time of the study was as a PhD student. A psychology student helped with minor chores. Questionnaire surveys have been useful in assessing why individuals select the bicycle as a means of transport [23,24,58,59]. Discussion groups were organized because the interaction among participants in a social context has been shown to enable the collection of less accessible data and insights [60]. Qualitative data from focus groups have informed similar transportation-focused issues in other places in the world [61]. 

For each of the 22 pictures of different bicycle-related environments projected onto the screen, participants were asked to imagine themselves bicycling on this facility and then indicate (Questionnaire available at: https://docs.google.com/document/d/1sH9nsmd-wrMSW7050nxHDlMSHhD_wv1u7MqVS1hkXlk/edit?usp=sharing) (Likert scale 0–6; 6 being the best and 0 the worst scenario) if this environment would:

(a) lower car/bike crashes
(b) lower crime
(c) increase economic development

Every image remained on the screen until each of the participants had ranked that picture on their survey. Immediately after everyone had ranked all the pictures individually (Phase One), members of this dinner-time group were shown the pictures again (Phase Two), but with the topic guide: “What do you see regarding the possibility of crashes, crime, and economic development?” For every picture, participants responded to the following questions:

(1) What aspect of the picture makes you think that it would lower or increase car/bike crashes?
(2) What element of the picture makes you think that it would lower or increase crime?
(3) What things in the picture give you the perception that it would increase/deter economic development?

All participants were encouraged to share their perception about the details in the pictures with the other group members, including saying what they liked or disliked about places to bicycle. All of the qualitative responses were audio recorded. Completion of the survey lasted around 30 min and group discussions lasted between 70 and 150 min. After completion of the surveys, 20 min or more remained to enjoy the dinner. The Michoacan State Commission on Bioethics revised and endorsed the research protocol.

### 2.4. Statistical and Content Analysis

Data were analyzed comparing differences based on gender, age and socio-economic status. To analyze crash, crime and economic development, we compared the means given to the images of cycle tracks with the means given to the other types of infrastructure using the *t*-test. *Pearson’s correlation* evaluated the correlation between images. The Student *t*-test was used to compare the variables between men and women, between participant ages 18 to 40 years or above 40 years old, and between participants with a socio-economic level less than or more than the median. *P*-value less than 0.05 was statistically significant. The Statistical Package for Social Sciences version 21 (SPSS Inc., Chicago, IL, USA) was employed.

Qualitative results describe features respondents thought would improve or deter safety, crime and economic development regarding places to bicycle. Transcriptions of the recorded comments allowed analysis of the participant’s perceptions. ATLAS.ti Software (Scientific Software Development GmbH, Berlin, Germany, 2012) analyzed the qualitative data. Of all the comments, only often-repeated and informative comments about changes to the built environment are included in a table with participant’s comments grouped under the respective headings for crashes, crime, and economic development. For the comments, design solutions with citations confirmed a similar finding. 

## 3. Results

Forty-three people (51% male; 18 to 61 years old—mean age 41, Table 1) participated in the six focus group dinners. All but one participant knew how to ride a bike, but only one bicycled on a daily basis. The first two dinners were held at neighborhood public schools (six and eight participants, everyone of a median socioeconomic level), the third was held at a public health community clinic (seven attendees, everyone under the median socioeconomic level), and the other three were at restaurants near where the participants lived (nine, six, and seven participants, all above the median socioeconomic level). The results of the quantitative data (6 best/0 worst scenario) (Phase One, survey) showed, from the mean of the scores, that cycle tracks had the highest score among all of the bicycle facilities for low crashes (4.56), low crime (4.14), and high economic development (4.33). For roads without bicycle provisions, men perceived lower chance of crash (0.69) compared with women (1.4). The results from the qualitative study (Phase Two, group discussions) showed that cycle tracks into which a car could be driven and/or parked (invadable cycle track due to low barriers) were not perceived as safe from crashes while cycle tracks into which cars could not be driven or on which cars could not be parked were perceived as safe. Participants perceived that cycle tracks into which cars could not drive or park helped to prevent crime and foster economic development.

### 3.1. Quantitative Analysis (Phase One, Survey)

The quantitative data revealed participant’s perceptions about cycle tracks versus the other bicycle facilities (Table 2). Cycle tracks were the most preferred bicycle facilities in relation to low crashes, low crime, and high economic development. Females (4.79) perceived the cycle tracks to be safer from crashes compared with males (4.35) and overall perceptions about cycle tracks were the highest compared with the other bicycle facilities. In the overall ranking of means for each image, the results suggested proper grouping of the images as they reflected that specific type of bicycle facility.

#### 3.1.1. Comparison of Means for Crash, Crime and Economic Development

Compared to cycle tracks, participants gave significantly higher rank for possibilities of crash and crime, and lower rank for economic development to the bicycle facilities that were less protected (invadable cycle track, bike lanes, bus and bike lanes, and roads). The ranking of cycle tracks was not significantly different for shared use paths under low crashes or for bike lanes under high economic development. The difference in gender for the perception of roads was statistically significant for bicycling in the road (1.4 for male versus 0.69 for female; *p* < 0.001). Females indicated higher crash possibility in roads with no bicycle facilities than men. Marginally significant differences existed between men and women concerning their perceptions of cycle tracks for improving economic development, with women indicating cycle tracks were more associated with economic development. No differences were found based on the age of participants (<50 and ≥50 years) or socio-economic level (<median and ≥median) (data not shown).

#### 3.1.2. Overall Ranking of Means

In the correlation between images, participants tended to equally rank these images: 1. One and fifteen (both cycle tracks protected with a wide area); 2. Three and nine (both with no bicycle facility); 3. Two and thirteen (both one-way streets with low traffic); 4. Seven and twelve (both bike lanes); 5. Fourteen and sixteen (the only ones with cycle tracks protected with parallel parked cars but no buffer); and 6. Eighteen and twenty-one (shared use paths and cycle track respectively) (Table 3). Thus, grouping of the images was correct.

### 3.2. Qualitative Analysis (Phase Two, Group Discussions)

These data were compiled based on the comments recorded when the participants viewed the pictures of bicycle environments. Comments about the six bicycle environments fell under category headings of crashes, crime, or economic development. Quotes selected included those that best captured the perception of the majority while divergent views demonstrated lack of consensus. The qualitative comments from the 43 participants included gender and age with specific attribution to 15 females and 15 males. Some participants were quoted more than once while some were not quoted because they agreed with the other person, i.e., when the other person spoke, they would say, “I think that too.” If more than one participant had the same characteristics, it was recoded to A, B and C (when necessary).

#### 3.2.1. Not Invadable Cycle Tracks

**Figure 3:**
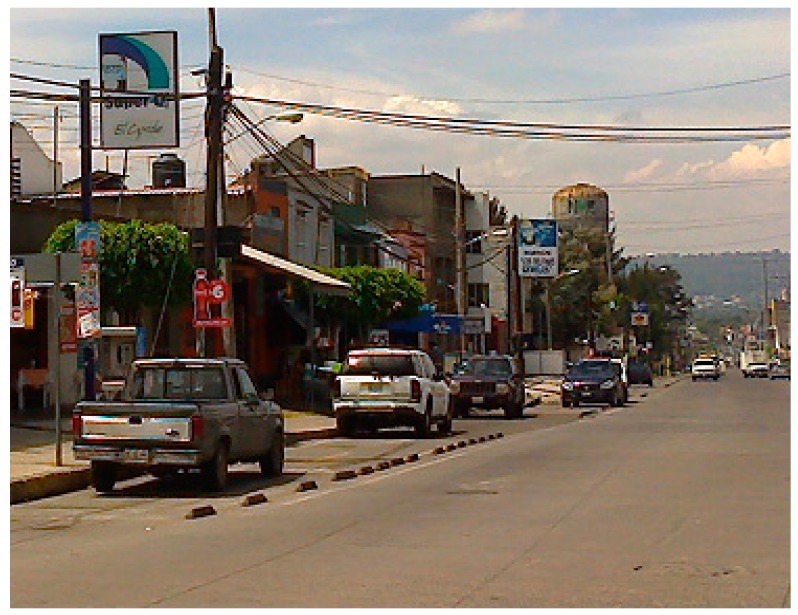
This image was not included in the survey. It shows how drivers park on a cycle track built in the city of Morelia around 2013.

Participant’s perception of the low possibility of crashes with automobiles on a cycle track into which a driver could not drive their car was associated with protection, due to the physical barriers such as concrete curbs and plants (Table 4A). Participants described this protection from vehicles with words including segregation, delineation, separation, containment, guard, and exclusive. Participants also pointed out that the less-able-to-be-driven-over (not invadable) physical barriers work better in a society where traffic rules are not obeyed (Figure 3). Almost all participants concluded that high quality bike infrastructure would invite them to use the bicycle as a mean of transport with one participant stating, “If there was this kind of bike infrastructure (so safe), I would use my bicycle for some utilitarian purposes” (female, 41 A). One participant also noted about cycle tracks: “The most important thing is that everyone knows where they should be” (male, 52 A). 

The participants consistently associated the absence of crime with the possibility of economic development (Table 4B). One participant said about the picture with the cycle track, “It’s secure because it is very busy; lots of people passing by” (female, 41 A). They linked a retail environment with a minor risk of delinquency, assuming that if the place attracts clients, then it is also a safe setting. Presence of retail, movement, and people were interpreted as environments with no or low presence of a felony. In reverse, participants perceived the lack of economic development fostered criminal behavior. 

Infrastructure with wide sidewalks and cycle tracks, along with visual amenities (trees, plants, shades, etc.) and people, elicited the perception that retail could thrive (Table 4C). Participants described the positive elements in the environment using descriptors including movement of people going by, beautiful sidewalks, open space, easy access, inclusive, well managed, and café tables. One comment summed up the economic promise in the design details, “Because of the wide sidewalks and place for people on bicycles, economic activity would do well” (female, 27).

#### 3.2.2. Invadable Cycle Tracks

The participants did not prefer invadable cycle tracks due to the possibility that car drivers could drive into or park on these cycle tracks. Individuals in other countries might respect a modest line on invadable cycle tracks, whereas, in Mexico, solid and high barriers are necessary to deter drivers. The participants indicated that separations needed to be large, visible, and not destructible. Even though these cycle tracks were invadable, security improved with the cycle track. For economic development, participants thought having ground floor retail was an economic trigger.

#### 3.2.3. Shared Use Paths

Shared use paths, similar to cycle tracks, were also associated with the perception of low car/bike crashes. However, compared to cycle tracks, shared use paths were not associated with lowering crime, in part because lack of lighting makes the area risky. Shared use paths were also not associated with an increase in economic development, because, as noted, the emphasis was on exercising and not shopping. On the other hand, perception of the ideal or permitted speed for those paths was polarized. Some perceived the path was a shortcut built to allow them to move at great speed while others thought calm enjoyment appropriate. For economic development, some believed that a few stores could flourish if they provided services to people on the pedestrian and bicycle path, but others mentioned that establishing business could be an intrusion in a place where people just want to exercise.

#### 3.2.4. Painted Bike Lanes

Painted bike lanes beside parallel-parked cars allow the possibility that a minor mistake, like opening a car’s door, could result in a negative consequence. Some participants argued that changing the order of the street users would improve the safety for people on bicycles, i.e., putting the bike lane beside the curb with parallel parked cars alongside (to form a barrier from moving cars, as in a cycle track according to CROW [56]. Painted bike lanes produced split opinions. Some of the participants determined that the paint was sufficient to ensure safety for bicyclists. Others insisted that no one respected a painted line and that the illusion of safety could invite new users, exposing them to imminent dangers (from door opening and or crashes with cars). Participants indicated that roads and painted bike lanes did not discourage crime. Participants also perceived that roads and streets with bike lanes are good for economic development, similar to cycle tracks, but people do not feel sufficiently safe riding a bike on them. According to the qualitative responses, infrastructure that privileges the car does not promote economic benefit, even though that is still a paradigm of urban planners in developing nations.

#### 3.2.5. Shared Bus and Bike Lane

When the pictures included streets and roads shared only by buses and bicyclists, participants agreed that the Mexican bus drivers would lack willingness to be courteous to bicyclists. They thought bicyclists would be at high risk for a crash. They also thought the bus and bike lane was a lonely place with high insecurity. When the participants discussed having a shared bus and bike lane downtown, they viewed the sharing and location favorably. They thought that a car-less landscape could improve the overall experience, prevent crime, and enhance economic activity.

#### 3.2.6. Road with No Bicycle Provision

Pictures with no bike-specific infrastructure elicited descriptors that included chaos, disorder, and conditions that would put a bicyclist at risk. Mentioned were uneven pavement, multiple automobile entrances and exits, interaction with public transit, and blind spots. Participants linked a place prone to crashes between bicycles and cars as “dangerous” and “risky.” Environmental features that included disorder, narrowness, and the possibility to invade a place did not match the needs of the vulnerable bicyclist. Limited space with narrow sidewalks gave participants the impression that there was an untapped potential in the distribution of space. Places with no bike infrastructure or poor sidewalks did not appear to be a place to shop, but only to pass by.

Participants identified the lack of signalization as unsafe for riding a bicycle. While symbols and traffic signs could indicate to users of urban infrastructure how to behave, repeatedly participants said that signs were not good enough and were not clear. The participants also perceived that, even if signs existed, individuals did not always heed the signs. 

On the roads with high traffic volumes, a participant (male, 35 A) observed that for people on bikes, “It is fundamental to have separated infrastructure.” One picture was of a local residential road and the participants thought the area presented latent risk. This perception differed from how they perceived high trafficked streets and where they sensed imminent risk. Other descriptors included the apprehension about a car door opening. Only one person mentioned that it would be necessary to put “cops on bikes” (male, 38) to enhance the security of the place, and another said that “environments would be safer if there were security cameras installed” (female, 56). For perceptions, crime was high due to the lack of people passing by while economic development was low because only cars were passing. One participant suggested that if there was order on the street, retail would succeed, and others agreed.

## 4. Discussion

Participants in Morelia, Mexico, viewed cycle tracks with solid barriers that cars cannot drive over or through as safer from crashes than invadable cycle tracks, painted bike lanes, bus and bike lanes or roads without bicycle provisions. Infrastructure should work without the need of law enforcement, such that any user—pedestrian, cyclist, or car driver—understands intuitively the right place to be and behaves accordingly. Cycle tracks were also associated with low crime and high economic development, in particular because there would be many people passing by. Compared with cycle tracks, participants perceived shared use paths safer from crashes but shared use paths offered few benefits for economic development or for lowering presence of crime, especially as they lacked street lighting. Bus and bike lanes were not as safe from crashes because of Mexican idiosyncrasy. While facilities such as invadable cycle tracks might work in developed nations because the drivers know to not drive into or park on the cycle track, in Morelia, drivers do not necessarily obey laws, signs, or lines. 

This research suggested that painted bike lanes, valued among industrialized countries [11,23,59,82,83] would only work in a developing nation, like Mexico, if reconstructed as cycle tracks with physical separations that prevent cars or trucks from entering or parking. Schoner, Cao and Levinson [84] stated that bicycle lanes attract existing bicyclists to a neighborhood, rather than inviting new users to adopt the bicycle as a way of transport. This research suggested that if bicycle environment designs provide optimal safety, people would feel invited to use that infrastructure, and be willing to shift from other modes. 

Female cyclists feel safest when separated from vehicles [16,18,23,24,27,85,86] and this study concurred because females ranked the cycle tracks higher for safety from crashes. The quantitative data indicate that women favor separation from vehicles, a finding that the women illustrate through their qualitative comments. Just 20% of the commuting bicyclists in Mexico are female [87] and the high percentage of comments from females in this study suggest that, in future studies, women should be targeted and shown pictures of different bicycle environments. Women would then have a voice about transportation. 

Urban infrastructure that considers all bicyclists’ needs is slowly emerging in developing nations like Mexico. Research conducted in developed nations already revealed that a lack of dedicated space for cyclists lowered the perceived level of safety, and limited the desire to ride [88]. The perception that bicyclists are safest when separated from traffic has been validated by others [89,90]. While in low-income countries cultural beliefs regarding the fatalism of road injuries prevail [90], perceptions can be challenged with the exposure to new ideas and possibilities. Participants of this study could see, imagine, and meditate about new approaches to address road safety. Road injuries can be deterred if the correct environment is provided [63]. Just as industrialized countries have revolutionized their road safety records by systematically improving their infrastructure, developing nations could follow suit by installing infrastructure that fits their culture and practices. The environment can coax good behavior, especially in a developing nation where driver and bicyclist training might not have been available to everyone. Self-enforcing measures, such as the physical separation provided by a cycle track, provides a more “forgiving roadside” [89] and is more likely to play an important role in the developing world. If cyclists have separate infrastructure and drivers their own exclusive lanes, road injuries decrease. 

Although Mexican media and politicians insist that the lack of security is solved by putting more police on the streets [91], this study suggested that participants feel safe from crime in certain environments, even when those environments show no police. Law enforcement is costly and subject to corruption, a phenomenon observed in Mexico. By enabling pedestrians and cyclists to use the public space could be a way to improve security, because of the “eyes on the street” and natural surveillance phenomena [33,35]. While in 1972 Donald Appleyard demonstrated that a street with high traffic curtailed interaction with neighbors across the street [92], with autonomous vehicles, Uber, Lyft, and transit, streets will continue to have vehicles. Perhaps the new crime-deterrent neighbors-out-socializing environment can be on each side of the street in the enhanced cycle track and sidewalk space. 

Even if a space feels populated with “autos going by,” some spaces can feel isolated, giving the perception of low economic development. If road width allows for car parking and cycle tracks, cycle tracks with solid barriers by parked cars could be good for business. A study in Australia indicated that a square meter of bike parking (bikes parked in racks on the sidewalk or in the space of one parallel parked car) resulted in $31 per hour in store purchases compared with $6 per square meter in car parking [50]. A protected bike lane installed along nine-blocks in the historic section of downtown Salt Lake City resulted in a sales increase of 8.7% compared with a 7% increase in sales throughout the city [48]. 

As Wright and Fulton [93] have written, in México, stakeholders have thought that non-motorized transport is counter to national aspirations. The focus has been on increasing the capacity of roads for vehicles because it is politically expedient, but the result has been more congestion and pollution. Prejudices and out-of-date urban planning practices still prevail among citizens. This paper shows that when people compare images of different built environments, they agree that high quality infrastructure for cyclists would be better for safety, security and local economy. This study suggests that people of diverse socio-economic levels are positive about the adoption of a new design model and non-cyclists are willing to turn the streets into cycling-friendly environments. 

Perceptions about bicycle infrastructure regarding the possibility of crashes, crime, and economic development are different in a developing nation perhaps because policy, institutional context, and policing (ticketing for unlawful parking) are not the same [94]. Almost all interventions and strategies that have been proven effective in high-income countries need to be evaluated in low-income countries with particular attention paid to the effectiveness of enforcement measures [90]. 

Even though climate change is not the main issue addressed by this research, it is undoubtedly now one of the biggest global concerns. “Transport policy decisions made today in developing nations will have profound ramifications on any possible attempt to control global greenhouse gas emissions” [93] (p. 692). Because of the old fleet, poor maintenance practices and limited vehicle testing, the impacts of motorization in developing nations are many times worse than an equal level of motorization in a developed nation. In third world nations, high levels of motorization result in high rates of injuries and deaths, and also high levels of pollution [95]. Cities should seize low carbon transport modes, together with reducing the need to travel in cities [96].

A shift in mode share is an effective way to lower the greenhouse gas emissions. A single percentage point reduction in motorized mode share and a subsequent gain by either non-motorized options or public transport is substantial in terms of greenhouse gas impacts [93]. Public transport and non-motorized modes are still dominant in developing cities, and road congestion is present at much lower levels of car ownership [94], all of which can be viewed as advantageous. But the poor conditions of the public transport, and the inadequate conditions for walking and cycling means that most developing-city citizens will move to private motorized vehicles as soon as they can afford it [93]. In fact, vehicle usage has been growing in México [97]. The challenge for cities is to improve their transport systems and build safer infrastructure for cyclists to preserve the market share of low-emitting modes and protect the most vulnerable populations. In cities with low income levels like Morelia [98], cycling, in particular, offers an equitable travel option for all [99].

Physical inactivity contributes to the pandemic of various chronic diseases [100] but in cities where physical activity is no longer needed in work or home, telling people to bike is not enough. Changing individual-centered behavior is also difficult [101]. The more successful alternative is to make the right changes to the built environment because the safest bicycle environments can exert a very powerful influence on the population without distinction of gender, socio-economic level, etc. In México City, the new protected cycle track on a main street (Paseo de la Reforma Avenue) and the bicycle sharing system “ecobici” increased the number of bicyclists on that road by 60%, including people who did not bicycle before [102]. If people feel safe, they will bicycle.

This paper focused on different types of bicycle route infrastructure but one location for higher risk is the intersection. While shared use paths typically do not cross intersections and sharrows and bike lanes place the bicyclists in the road already, on a cycle track the barrier ends and the bicyclist is vulnerable to being hit by drivers making turns or crossing the intersection. The city of Montreal installed a network of cycle tracks twenty years ago and a recent analysis in Montreal showed that, as the bicyclist crossed the intersection, the bicyclist on a cycle track that crosses the intersection on the right or left is safer than the bicyclist on a road without a cycle track [103]. New York City installed cycle tracks within the past ten years and, on streets with bicycle facilities including the intersection, there were twenty-two bicyclist fatalities with five on a street with a cycle track, eleven on a street with a painted bike lane, and six on a street marked (sharrow) or signed [104].

Cyclist collisions are sensitive to changes in both cyclist and motor vehicle flows. Right-turn movements have a great effect on injury occurrence. The number of bus stops in the proximity of the intersection are prone to increase cyclist injury occurrence [103]. Therefore, developing nations interested on improving this mode share of transport should look for the best practices in the world [56,105,106,107]. Not all streets need or have the width for a cycle track so planners need to consider the range of safe cycling facilities. 

## 5. Limitations

The sample size achieved of 43 was only 22% of the original planned research protocol (*n* = 200). Given the free dinner, the response rate was, at a minimum, expected to be close to 50%. Participants who did attend indicated that the low response rate may have been due to uncertainty about the purpose, general mistrust about surveys, apprehension that the stakeholders would take advantage of people, and fear of going out at night because the city (and in general, the state) had high rates of crime. More mailings may have increased the sample but, after learning about participant’s fear of going out at night, the conclusion was the risk was too great. The comments by later group members also indicated that there was no need to continue with more visual and verbal preference survey groups because the respondents had been describing the images with similar words and descriptive examples (all cited in Table 4). Participants of all ages and from the two socio-economic levels had been giving similar qualitative responses and marking quantitative responses that were not highly variable. Even though bias could be present in the quantitative analysis due to the small sample size, the strength of this research was in the qualitative data. The greatest finding was that the responses in a developing nation were different from those in developed nations, as revealed in the insightful qualitative comments. 

## 6. Conclusions

While other studies have already determined that cycle tracks are preferred and safest, this is the first study to show that residents in a developing nation perceive cycle tracks as safest but that the designs must be different from those in a developed nation. Painting bike lanes or installing affordable plastic delineator posts is termed by transportation planners to be “low hanging fruit” but building less safe facilities in a new-to-the-bike country risks the lives of bicyclists. Many of the early bike adopters in a developing nation are lower income individuals who do not own cleat-equipped and lightweight bikes to weave their way out of danger. Only in developed nations where there are high levels of ticketing, policies to support bicycling, and social norms that hold bicyclists in high regard, should bicyclist share lanes with cars and buses. 

In a developing nation, solid-barrier cycle tracks should be located to guarantee high numbers of bicyclists. Having a treacherous bike route to a cycle track would mean that few would ride in the new separated bike facility. Then, critics would suggest eliminating the cycle track due to low use. If, in a developing nation, cycle tracks only have a slightly raised cobblestone divider, as in The Netherlands, and drivers swerve into or park in these cycle tracks, critics could then say that the non-functioning cycle tracks should be removed and the lane given back to vehicles. Therefore, in a developing nation the first cycle tracks should be in locations that would guarantee high use, such as a connector from a highly frequented shared use path to a Main Street area with eateries. This cycle track should also have a high solid barrier so drivers cannot enter. 

As organizations and researchers are putting the bicycle on Mexico’s national agenda, now is the time to establish laws and construction policies that foster cycling as a way of life, especially due to the demonstrated benefits of lowering crime and improving the economy. The results from this study in Morelia could be applied all over Mexico because Mexican cities present more or less the same issues related to health, pollution, road injuries, mobility problems, and transport policy deficiencies. Cities in Mexico are compact compared with cities in the USA or Canada that are sprawled and have considerable travel distances [45]. Even for most sprawled city in Mexico, Mexico City, the average trip distance is still only 9.9 km. In Morelia, (among the 20th biggest cities in Mexico), 50% of trip distances are less than 3 miles [3], suggesting that around fifty percent of the trips could be by bike.

In Mexico, public policies for health and mobility should be complementary because of the shared goals of serving individuals and the cities. Transport and urban design, both of which involve land use and housing policies that make walking and cycling possible, should be transversal with actionable items accomplishable in all the cities. Though papers on improving cycling in Latin American countries and in Mexico are available and the World Resources Institute [108], the Institute for Transportation and Development Policy [109], and the Sustainable Urban Transport Project (SUTP) [110] have supported biking, far more needs to be done in Mexico to increase biking in all populations.

This study revealed that cycle tracks are perceived as safer but also capable of lowering crime and increasing economic development. Because crime and economic development are vexing problems in Mexico, achieving multiple goals might be worth the cost of the cycle track. Participants summed up the value of the cycle track for safety, crime, and economic development in a developing nation by pointing that everyone would understand intuitively the order of the space. 

## Figures and Tables

**Table 1:** Participant’s demographics.

		Socioeconomic Level	
Age	Sex	Under Median	Above Median
18–25	Male	0	0
	Female	2	0
26–35	Male	0	8
	Female	2	4
36–45	Male	5	1
	Female	4	2
46–55	Male	4	2
	Female	1	2
56 and older	Male	1	1
	Female	2	2
		21	22

**Table 2:** Means given to the images, grouped by type of infrastructure on the core areas: Crashes, Crime, and Economic Development (Phase One).

		Comparisons of Means between Cycle Tracks and other Types of Infrastructure			Intragroup Comparisons of Means between Gender					
		A. Low Crashes	B. Low Crime	C. High Economic Development	A. Low Crashes		B. Low Crime		C. High Economic Development	
					Male	Female	Male	Female	Male	Female
	N	43	43	43	22	21	22	21	22	21
Cycle tracks	Mean ± SD	4.56 ± 1.00	4.14 ± 0.819	4.33 ± 0.77	4.35 ± 1.008	4.79 ± 0.97	4.16 ± 0.655	4.12 ± 0.978	4.1 ± 0.77	4.56 ± 0.717
	*p* value					0.157		0.876		0.053	
Invadable cycle tracks	Mean ± SD	3.78 ± 1.1	3.31 ± 0.89	3.06 ± 1.02	3.86 ± 1.047	3.69 ± 1.187	3.28 ± 1.014	3.34 ± 0.785	3.28 ± 0.938	2.84 ± 1.08
	*p* value	**0.000**	**0.000**	**0.000**	0.631		0.826		0.157	
Shared use paths	Mean ± SD	4.470 ± 1.16	2.960 ±1.26	2.43 ± 1.45	4.4 ± 1.19	4.54 ± 1.17	2.88 ± 1.34	3.04 ± 1.203	2.02 ± 1.248	2.85 ± 1.566
	*p* value	0.552	**0.000**	**0.000**	0.703		0.681		0.060	
Bike lanes	Mean ± SD	1.750 ± 1.06	2.93 ± 1.07	3.74 ± 1.11	1.86 ± 0.912	1.65 ± 1.217	2.83 ± 0.912	3.04 ± 1.244	3.81 ± 0.968	3.65 ± 1.26
	*p* value	**0.000**	**0.000**	0.006	0.519		0.522		0.645	
Bus and bike lane	Mean ± SD	1.68 ± 0.99	3.0 ± 0.93	3.38 ± 1.04	1.86 ± 0.875	1.5 ± 1.09	3.0 ± 0.771	3.02 ± 0.164	3.31 ± 0.852	3.45 ± 1.23
	*p* value	**0.000**	**0.000**	**0.000**	0.235		0.935		0.679	
Roads with no bicycle provision	Mean ± SD	1.05 ± 0.73	2.42 ± 0.78	2.71 ± 1.06	1.4 ± 0.704	0.69 ± 0.577	2.4 ± 0.745	2.36 ± 0.834	2.91 ± 1.03	2.5 ± 1.08
	*p* value	**0.000**	**0.000**	**0.000**	**0.001**		0.657		0.208	

Statistically significant comparisons are shown in **bold**. IC (99%). Mean: 6 is the best and 0 the worst scenario.

**Table 3:** Overall ranking means given to the images, by type of infrastructure. Comparisons between groups of images (Phase One).

Type of Infrastructure		Compared To		
	Mean ± SD		Mean ± SD	*p*
Cycle tracks	4.3473 ± 0.622	Invadable cycle track	3.39 ± 0.749	**0.000**
		Shared use paths	3.29 ± 0.94	**0.000**
		Bike lanes	2.81 ± 0.767	**0.000**
		Bike and bus lanes	2.69 ± 0.653	**0.000**
		Road w/no bike prov	2.06 ± 0.589	**0.000**
Invadable Cycle tracks	3.39 ± 0.749	Shared use paths	3.29 ± 0.94	0.503
		Bike Lanes	2.81 ± 0.767	**0.000**
		Bus and bike lane	2.69 ± 0.653	**0.000**
		Road w/no bike prov	2.06 ± 0.589	**0.000**
Shared use paths	3.29 ± 0.94	Bike Lanes	2.81 ± 0.767	**0.003**
		Bus and bike lane	2.69 ± 0.653	**0.001**
		Road w.no bike prob	2.06 ± 0.589	**0.000**
Bike Lanes	2.81 ± 0.767	Bus and bike lane	2.69 ± 0.653	0.323
		Road w/no bike prov	2.06 ± 0.589	**0.000**
Bus with bike lanes	2.69 ± 0.653	Road w/no bike prov	2.06 ± 0.589	**0.000**

Statistically significant comparisons are shown in **bold**. IC (99%). Mean: 6 is the best and 0 the worst scenario.

**Table 4:** Comments of participants regarding the types of infrastructure on the core areas (Phase Two): A. Crashes, B. Crime, C. Economic development, and design solution.

	Comments	Design Solution
**A. Crashes**		
Cycle track	“The most important thing is that everyone knows where they should be.” (male, 52 A) “There is a right place for each one.” (female, 49) “If there was that kind of bike infrastructure (so safe), I would use my bicycle for some utilitarian purposes”. (female, 41 A)	Physical separation that makes evident the place for each user of the road, regardless of level of education [56]. Bicycle facilities physically separated from cars and bicycle exclusive paths [62,63]. Robust bicycling infrastructure to increase the preference of cycling [64,65].
	“Where everyone would want to stay.” (female, 25) “A paradise.” (female, 60) “I think the curb or the planter is the best. They cause more respect than the bumpers which can be crushed or jumped with the car.” (male, 39) “(the place)…is not 100% safe, but there is a curb that separates them (people on bicycles) from the cars.” (female, 27) “…(plants) bring the feeling of a real division.” (male, 54)	A place to enjoy, rather than to go by [12]. Trees and or plants are not an amenity, they’re a necessity [66].
	“Seems like they give importance and respect to the bicycle path.” (female, 47)	Build cycle tracks that provide a sense of equity for all users; cycling infrastructure that is not only safe, but also convenient and attractive [41].
Invadable cycle track	“I prefer thousand times the curbside.” (female, 54) “There is a risk with the parked cars because of the door openings.” (male, 29)	Leave a buffer between the cycle track and the parked cars. Use barriers that impede the temporary invasion from the automobiles [56].
	“Cars don’t respect the cycle track.” (male, 44) “Drivers would invade it during the parking maneuvers.” (male, 40) “There’s no road culture at all. They don’t respect the signals and park everywhere.” (male, 63) “The lack of consciousness from the automotive drivers, makes them constantly invade diverse sections of the cycle track.” (female, 34) “That could be possible in another country, with a better culture.” (male, 31) “It is really important to have physical delimitations, barriers.” (female, 34) “In México, people ought to have a barrier, because if not, drivers would invade, with all sorts of excuses.” (female, 54)	Physical separation can be a great substitute if law enforcement is absent and/or if people tend to disobey traffic signals.
	“There should be bigger protections with more visibility and lower ability to be destroyed.” (male, 40) “…protection dividing parked cars and cycle track.” (female, 28)	Metal fences can be aesthetic, durable, and easy removable when needed (for instance, to widen the cycle track).
Shared use path	“Bicycle can cause an accident with pedestrians” (male, 61) “(when sharing the space with cyclists) …especially kids and pets are vulnerable.” (female, 28) “Made for a Sunday.” (female, 36) “For recreational purposes.” (male, 33)	Provide separate paths for pedestrians and cyclists [67]. Expect them to be used on weekends.
Painted bike lane	“I think it is poorly designed because the cyclist is placed after the parked cars, beside the vehicle flow.” (female, 56) “Cars could use the cycle lane to pass on the right.” (male, 39)	Protected cycle tracks.
	“The separation does not work with paint.” (female, 37) “The car driver invades the lane meant to be for the cyclist.” (female, 56) “The illusion of safety could invite new users, exposing them to imminent dangers.” (male, 40)	Physical clear separation is needed in order to provide a safe ride. Let the infrastructure forgive possible mistakes of users of the road.
Bus and bike lane	“With Mexican idiosyncrasy, you cannot expect bus drivers to share the road.” (female, 49)	Separate bus lane from bike lane: protected cycle tracks.
Road with no bicycle provision	“The cyclist is fully exposed to an accident.” (female, 37) “(The person on a bicycle) … is playing with his/her life.” (female, 60) “It is hard that a child or a woman takes the risk ridding through that avenue, unless it is strictly necessary.” (female, 41 B) “It is fundamental to have a separated infrastructure.” (male, 35 A)	Focus built environment on safety for all users (pedestrian, cyclists, transit riders, etc), rather than speed of vehicles [68].
**B. Crime**		
Cycle track	“It’s secure because it is very busy; lots of people passing by.” (female, 41 A)	The fear of crime can be reduced in places where there are people (pedestrians, cyclists) passing by and activity taking place [69,70]. “Eyes on the street” and “natural surveillance” and fixing broken windows [33,35,46]. Social fabric that defends itself [45]. Places free from crime and from the fear of crime improves the quality of life [34,69,71].
	“I see it is a secure place, because trees are trimmed. It allows one to see far away. There’s lighting.” (male, 52 B)	Tree species should be carefully chosen and trimmed to increase visibility.
	“Bushes are sometimes used to hide to assault.” (male, 40) “Trees give the impression of insecurity during night.” (female, 36)	Use short bushes.
Invadable cycle track	“Security is improved with the cycle track (people going by).” (female, 42)	Make the cycle track attractive. Well-developed local network structures reduce crime by increasing informal control [72].
Shared use paths	“There is little public lighting and it becomes highly risky.” (female, 33)	Provide sufficient public lighting.
Painted bike lane	“Plenty of insecurity, because there are a lot of parked cars, and they can become a place for someone to hide and assault.” (male, 33)	Use parking spaces to build a cycle track and/or widen the sidewalk.
Bus and bike lane	“It seems like a very lonely place. There is high insecurity.” (female, 41 C)	
Road with no bicycle provision	“There can be crime because there are no people around.” (female, 36) “Delinquency is looking for lone places to perform.” (male, 52 B) “I am alarmed by the possibility of crime, because it seems a low transited zone.” (female, 54)	Improve pedestrian and cyclists flow by widening the sidewalks and building safe bike infrastructure [37]. Busy streets lower vulnerability compared with not busy.
**C. Economic development**		
Cycle track	“Economic development does great, and therefore there is less risk of crime.” (female, 60)	Build places that will attract people (wide sidewalks, trees and plants, benches). Bike lanes and on-street parking have been found to increase business [49,73,74].
	“There is economic development because there’s a lot of movement, a lot of people going by.” (female, 33)	Invest in bicycling infrastructure. “It is a cost-effective way to enhance shopping districts and communities, generate tourism and support business” [49] (p. 2)
	“There’s a lot of potential for economic development, because of the wide sidewalks.” (female, 42) “Because of the wide sidewalk, and places for people on bicycles, economic activity would do well.” (female, 27) “There is a beautiful sidewalk that allows one as a pedestrian to want to walk on that place.” (male, 39)	Widen sidewalks [13].
	“It is a public open space. One can sit down. It could increase retail revenues. One craves for something.” (male, 35 B)	A place to enjoy, rather than to go by.
	“Economic development is favored. Easy access.” (female, 41 C) “It is a very inclusive area, of every users of the street.” (female, 36) “Spaces are well managed, distributed among all users of the road.” (male, 42)	Build infrastructure that provides a sense of equity for all users [75,76].
	“I love cafe tables outside. The foreigner likes to sit under the sun, be in the outdoors and feel the city.” (female, 54)	Allow cafes and restaurants to have tables outside.
Invadable cycle track	“Ground floor for retail is an economic trigger.” (female, 53)	
Shared use path	“There is no possibility of economic development because it is a way for people to exercise.” (male, 35 B)	Even though retail is not fostered by this type of infrastructure, it is of value itself [77].
Painted bike lane	“It is a lonely place. Retails wouldn’t do well.” (female, 60)	Promote mixed land use.
Bus with bike lanes	“It would be very positive for downtown development, so that people could walk calmly. It looks better without cars.” (female, 54)	When there is little space, use bus-bike and sharrows together with traffic calming strategies [78,79,80].
Road with no bicycle provision	“Low economic development, because there are just cars going by.” (male, 33) “Retail can benefit if access to them were diverse.” (female, 41 B) “Adequate infrastructure would allow profit for retail.” (female, 28) “Economic development could be improved by better administration of the public space, because it is now neglected.” (male, 33) “I think that retail would do well because of the location but poor accessibility could affect business. There is no order on the street.” (male, 61)	Built infrastructure that provides accessibility for all users. Increase in bicycling can be of great economic impact [81].
